# Proteomic Profiling Reveals Upregulated Protein Expression of Hsp70 in Keloids

**DOI:** 10.1155/2013/621538

**Published:** 2013-10-24

**Authors:** Ju Hee Lee, Jung U. Shin, Inhee Jung, Hemin Lee, Dong Kyun Rah, Jin Young Jung, Won Jai Lee

**Affiliations:** ^1^Department of Dermatology and Cutaneous Biology Research Institute, Yonsei University College of Medicine, 50 Yonseiro, Seodaemun-gu, Seoul 120-752, Republic of Korea; ^2^Department of Plastic and Reconstructive Surgery, Institute for Human Tissue Restoration, Yonsei University College of Medicine, 50 Yonseiro, Seodaemun-gu, Seoul 120-752, Republic of Korea; ^3^Yeouido Oracle Cosmetic & Dermatologic Surgery Clinic, Seoul, Republic of Korea

## Abstract

*Background.* The biochemical characteristics of keloid-derived fibroblasts differ from those of adjacent normal fibroblasts, and these differences are thought to be the cause of abnormal fibrosis. Therefore, we investigated the characteristic proteins that are differentially expressed in keloid-derived fibroblasts using proteomics tools. *Objective.* We attempted to investigate the novel proteins that play important roles in the pathophysiology of keloids. *Methods.* Proteomics analysis was performed to identify differentially expressed proteins in keloid-derived fibroblasts. Keloid-derived fibroblasts and adjacent normal fibroblasts were analyzed with 2-DAGE. We validated these proteins with immunoblot analysis, real-time RT-PCR, and immunohistochemistry. *Results.* Sixteen differentially expressed protein spots were identified in keloid-derived fibroblasts. Among them, heat shock protein 70 (Hsp70) was specifically upregulated in keloid-derived fibroblasts. Also, immunohistochemistry and western blot analysis revealed increased Hsp70, TGF-**β**, and PCNA expressions in keloids compared to normal tissue. *Conclusion.* Hsp70 is overexpressed in keloid fibroblasts and tissue. The overexpression of Hsp70 may be involved in the pathogenesis of keloids, and the inhibition of Hsp70 could be a new therapeutic tool for the treatment of keloids.

## 1. Introduction

Keloids are pathologic proliferations of the dermal layer of the skin that result from excessive collagen production and deposition. With respect to their pathogenesis, various explanations including ischemic [1], mechanical [2], hormonal [3], autoimmune [4], and genetic theories [5] have been suggested. In keloids, the homeostasis of wound healing is not maintained, resulting in excessive synthesis of extracellular matrix components such as collagen, fibronectin, elastin, and proteoglycans [6–8]. Additionally, compared to normal dermal fibroblasts, keloid fibroblasts react differently to metabolic factors that regulate apoptosis [9], extracellular matrix metabolism, glucocorticoids, growth factors [10, 11], and phorbol esters [12]. These abnormal fibroblasts have been considered to be the cause of the abnormal scarring that occurs with keloids, hypertrophic scars, and pathologic organ fibrosis. 

Proteomics can be used to separate proteins by two-dimensional electrophoresis (2-DE) and to characterize proteins using several analytical tools. The major advantage of this proteomic technology is that it allows for the analysis of the whole protein and studies differentially expressed protein instead of analyzing each individual proteins separately. Therefore, studies in the complete proteome level can lead us to characterize and understand the unknown events involved in the biological process. With this advantage, proteomics have been recently used in wide range of dermatologic field, such as aging, cancer, and UV influence. Two separate studies previously investigated keloid tissue and normal skin to compare their protein profiles [13, 14]. In this study, we compared primary cultured fibroblasts from keloid tissue and normal skin instead of comparing tissue extracts. We hypothesize that different protein expression profiles in keloid fibroblasts can provide novel information of keloid pathogenesis. By comparing fibroblasts, we attempted to characterize keloid fibroblasts specifically from normal fibroblasts. Then, we confirmed the expression of keloid fibroblast-specific proteins using immunohistochemistry, western blots, and quantitative RT-PCR. 

## 2. Materials and Methods

### 2.1. Patients and Sample Collections

After obtaining informed consent according to a protocol approved by the Yonsei University College of Medicine Institutional Review Board, keloid tissues were obtained for fibroblast culture and immunohistochemistry with excision. Keloid fibroblasts and normal fibroblasts were obtained from both the central dermal layer of keloids and the adjacent normal dermis from patients with keloids in the active stage (Table 1). All experiments involving humans were performed in adherence with the Helsinki Guidelines. Keloids were identified by trained clinicians and pathologists.

### 2.2. Fibroblast Culture

Primary fibroblasts and HDF cells (PCS-201-010, ATCC, Manassas, VA, USA) were cultured in Dulbecco's modified Eagle's medium (DMEM; GIBCO, Grand Island, NY, USA) and supplemented with heat-inactivated 10% fetal bovine serum (FBS), penicillin (30 U/mL), and streptomycin (300 *μ*g/mL). When the primary culture became confluent, the culture medium in the primary culture flasks was removed, and the cells were washed with PBS, detached completely by adding 2 mL of 0.025% trypsin, and then collected. All cells used in this study were collected before Passage 5. 

### 2.3. 2D Electrophoresis

Keloid fibroblasts and normal fibroblasts were washed with PBS, collected with a cell scraper, centrifuged, and then the pellets were collected. Pellets were diluted with sample buffer containing 7 M urea, 2 mM thiourea, 4% 3-[(3-cholamidopropyl)dimethylammonio]-1-propanesulfonate (CHAPS), 2 mM tributylphosphine (TBP), 0.5% carrier ampholytes, 40 mM Tris, and 0.001% bromophenol blue (BPB). An immobilized pH gradient (IPG) was created with pH 4–7 strips that were rehydrated and was added to a sample cup. Then, 350 *μ*L of each sample was centrifuged and loaded onto the strip. To prevent recrystallization of urea, 2 mL of covering fluid was added, and the samples were rehydrated at room temperature for approximately 12 hours. The samples were put onto the IPG strips using an IPGphor isoelectric focusing (IEF) apparatus (Amersham Pharmacia Biotech, Piscataway, NJ, USA) at 20°C. The IEF was conducted at up to 8000 V for 64 kVh. After IEF, each strip was shaken lightly in 20 mM Tris-HCl (pH 8.8) containing 6 M urea, 2% SDS, 20% glycerol, 2.5% acrylamide, and 5 mM TBP for 15 minutes to equilibrate samples for SDS-PAGE. The gradient of polyacrylamide gel (23 × 20 × 0.1 cm) used in this experiment was 8–16%. Each IPG strip containing a focused sample was placed on a gel and sealed in place with SDS containing 0.5% low melting point agarose and 0.001% BPB. Electrophoresis was performed using ISO-DALT equipment (Hoefer, San Francisco, CA, USA) for approximately 19 hours at 100 V until the dye reached the bottom of the gel. The gel was destained with 10% methanol and 7% acetic acid for 4 hours, then the gel image was scanned using a GS-800 calibrated densitometer (Bio-Rad, Munich, Germany). The scanned images were analyzed using Melanie III 2D electrophoresis analysis software (ver. XX, Bio-Rad). 

### 2.4. *In Situ* Digestion and MALDI-TOF MS

After excising the protein spots from the gels, the spots were washed with 25 mM ammonium bicarbonate (pH 7.8) and 50% acetonitrile (ACN) solution and dried using a SpeedVac evaporator. For each protein sample containing gel residue, 10 *μ*L of 0.02 g/L trypsin was added, and the mixture was incubated on ice for 45 minutes. Then, 50 mM ammonium bicarbonate buffer was added again (pH 7.8), and the mixture was incubated at 37°C for 12–14 hours. To facilitate the extraction of peptides, 10 *μ*L of 0.5% TFA and 50% ACN were added, and the mixture was sonicated 3 times for 10 minutes. The extracted peptide samples were spotted on a target plate and completely dried at room temperature. MALDI-TOF MS results were obtained on a Voyager-DE STIR mass spectrometer (PerSpective Biosystems, Framingham, MA, USA). Peptide matching and protein searches against the NCBI nonredundant protein database were performed using the ProFound program (http://prowl.rockefeller.edu). For screening the search data, only peptides yielding larger than a minimum Est'd Z score of 1.645 were accepted as having extensive homology.

### 2.5. Western Blot Analysis to Evaluate Hsp70

Cells were transferred to a micro centrifuge tube and centrifuged for 2 minutes at 3000 rpm. The remnant cells were used as a cell lysate. RIPA buffer (10 mM PBS, 1% NP40, 0.5% sodium deoxycholate, 0.1% SDS) containing proteinase inhibitors (10 mL/mL PMSF (10 mg/mL), 30 mL/mL Aprotinin (Sigma, cat# A6279) and 10 mL/mL sodium orthovanadate (100 mM)) was added, and the cells were lysed. We then added electrophoresis loading buffer (1.0 mL glycerol, 0.5 mL 2-mercaptoethanol, 3.0 mL 10% SDS, 1.25 mL 1.0 M Tris-Hcl (pH 6.7), 1-2 mg BPB) to the samples and boiled the samples for 3 minutes. After 10% SDS-PAGE, the proteins were transferred to a nitrocellulose membrane (Millipore Co., Bedford, MA, USA) for 2 hours in a container filled with transfer buffer solution. The membrane was blocked with 5% normal fibroblasts at skim milk in 10 mM Tris buffered saline (TBS, pH 8.0) containing 0.05% Tween 20, and antibody against Hsp70 (Stressgen, BC, Canada), which was diluted to 1 : 1000, was added. The membrane was then thoroughly washed, and immunocomplexes were detected using an enhanced horseradish peroxidase/luminol chemiluminescence system (ECL Plus, Amersham International plc, Little Chalfont, UK), followed by autoradiography (Hyperfilm ECL, Amersham International plc). Immunoblot signals were quantified using a computer imaging program (GelScopeTM, Imageline Inc., CA, USA).

### 2.6. Quantitative Real-Time Reverse Transcription-Polymerase Chain Reaction (qRT-PCR)

Total RNA was prepared with the RNeasy Mini Kit (Qiagen, Germany), and complementary DNA was prepared from 1 *μ*g of total RNA by anchored-oligo (dT)_18_ primer using a Transcriptor First Strand cDNA Synthesis Kit (Roche, Germany) under the following conditions: 65°C for 10 min, 50°C for 1 hr, and 85°C for 5 min. Applied Biosystems Taqman primer/probe kits (assay ID: Hs00359163_s1∗ (Hsp70)) were used to analyze mRNA expression levels with the use of an Applied Biosystems 7500 Real-Time PCR System (Applied Biosystems, Foster City, CA, USA). Target mRNA levels were measured relative to an internal glyceraldehyde-3-phosphate dehydrogenase (GAPDH) control (assay ID: Hs99999905_m1, Applied Biosystems). For cDNA amplification, AmpliTaqGold DNA polymerase was activated after 2 min incubation at 50°C followed by activation at 95°C for 10 min; this was followed by 50 cycles of 15 sec activation at 95°C and 1 min at 60°C for each cycle. To measure cDNA levels, the threshold cycle at which fluorescence was first detected above baseline was used, and a standard curve was drawn between the starting nucleic acid concentrations and the threshold cycle. The mRNA expression levels were normalized to the levels of GAPDH housekeeping genes. Each experiment was performed three times.

### 2.7. Immunohistochemical Assessment in Tissues

Immunohistochemistry (IHC) was performed in 10% formalin-fixed, paraffin-embedded keloid tissues. Tissues were pretreated with a 3% hydrogen peroxide solution for 10 minutes, washed again with DW several times for 10 minutes each, and incubated with 1xTBST (TBS + 0.1% Tween 20) for 5 minutes. Tissues were then treated with normal goat serum (Vector Laboratories, Inc., USA) at room temperature for 1 hour to prevent nonspecific reactions and incubated overnight with mouse anti-Hsp70 (ab6535, Abcam, UK) antibody and anti-TGF-*β* (GTX110630, Gene Tex Inc., San Antonio, TX) diluted to 1 : 100. The tissues were then washed with 1xTBST and incubated for 30 minutes at room temperature with biotinylated secondary antibody solution from the Dako REAL EnVision Detection System (Dako, Denmark), washed with distilled water, counterstained with hematoxylin (SIGMA-ALDRICH, Inc., USA), dehydrated and clarified by a conventional method, and prepared for examination under a light microscope. The expression levels of Hsp70 and TGF-*β* were semiquantitatively analyzed using MetaMorph image analysis software (Universal Image Corp.). Results are expressed as the mean optical density of six different digital images.

### 2.8. Statistical Analysis

The results obtained were analysed by paired *t*-test and one-way ANOVA. All data were summarized as mean ± standard deviation (SD). Deviations were considered statistically significant when *P* < 0.05. 

## 3. Results

### 3.1. Heat Shock 70 kDa Protein 1A Was Upregulated in Keloid Fibroblasts

**Figure 1:**
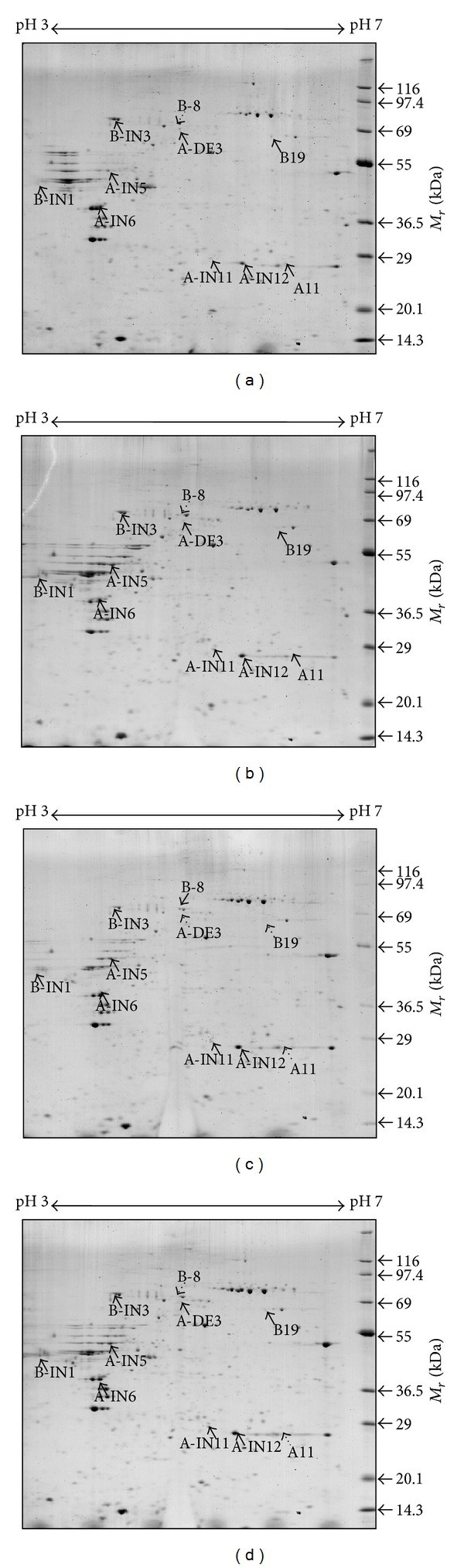
2DE proteomic profiles of total protein extracts from normal fibroblasts (a, b) and keloid fibroblasts (c, d). Alphabets and numbers indicate proteins identified by MALDI-TOF and ProFound program. Further information on each protein is provided in Table 2.

Using primary cultured keloid fibroblasts and normal fibroblasts from two patients, we extracted total protein, completed 2-DE within the pH 4–7 linear range. Approximately 800 spots were revealed by image analysis with Melanie III. Using MALDI-TOF MS, we identified final 16 protein spots which were consistently increased or decreased in both patients (Figure 1, Table 2). Heat shock 70 kDa protein 9B (also known as Hsp70-9B or mortalin), vimentin, dnaK-type molecular chaperone HSPA1L (also known as Heat shock 70 kDa protein 1L, Hsp70-1L), and dihydropyrimidinase-like 2 were only detectable in keloids and crocalbin-like protein, calumenin, vimentin, BiP (also known as Hsp70-5, GRP78), tropomyosin 1, and heat shock 70 kDa protein 1A (also known as Hsp70-1A) were upregulated in keloids. Among these proteins, several heat shock proteins (HSPs) were found to be differentially expressed in keloid fibroblasts, and upregulated HSPs were all Hsp70 families. 

### 3.2. Overexpression of Hsp70 in Keloid Fibroblasts Was Confirmed by qRT-PCR and Western Blot

**Figure 2:**
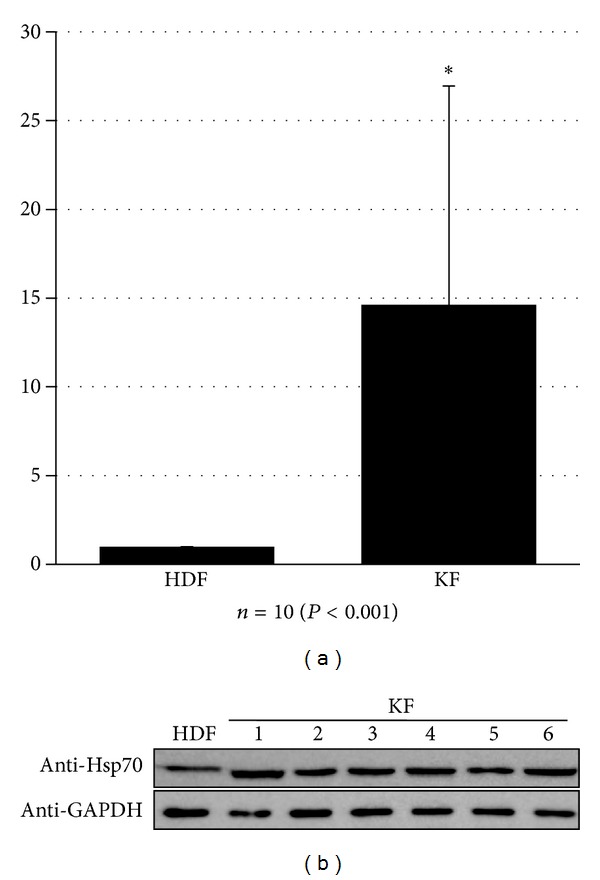
Identification of Hsp70. (a) The expression of Hsp70 mRNA was specifically elevated in keloid fibroblasts. (b) Increased Hsp70 in keloid fibroblasts was confirmed by western blot analysis.

In primary keloid fibroblasts, the mRNA expression of Hsp70 was examined using real-time reverse transcription-polymerase chain reaction (RT-PCR). The quantitative analysis indicated that the Hsp70 mRNA level in keloid fibroblasts was markedly increased (*P* < 0.05) (Figure 2(a)) compared to HDF. Also, immunoblot data showed that protein level of Hsp70 was significantly increased in keloid fibroblasts compared to the HDF (Figure 2(a)). Both RT-PCR and western blot showed that all keloid fibroblasts highly expressed Hsp70 than HDF. From these results, we found that Hsp70 is overexpressed in all keloid fibroblasts in mRNA and protein level.

### 3.3. Hsp70 Protein Expression Was Significantly Elevated in Keloid Tissues

**Figure 3:**
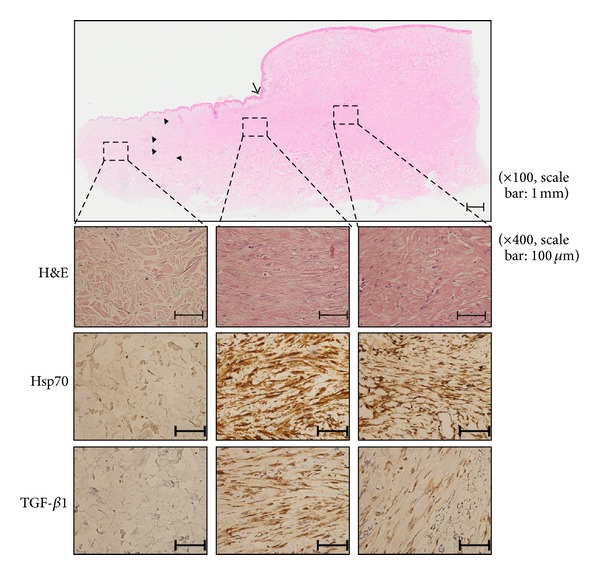
Immunohistochemical staining of keloids and adjacent normal dermal tissues. Abnormal dense collagen fibers (arrow heads) were extending over clinical keloid margin (arrow). Hsp70 and TGF-*β* were also increased along with extending collagen fibers (×100 and ×400).

**Figure 4:**
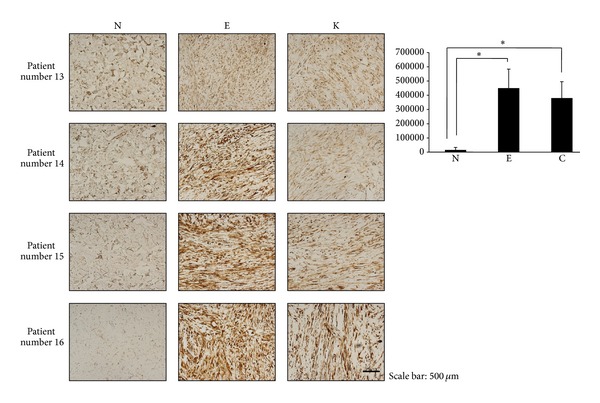
Quantitative analysis of Hsp70 protein expression. The immunohistochemical study (*n* = 4) showed that Hsp70 expression within the deep dermal layer of keloid tissues was higher than in the normal adjacent dermis. The increased expression of Hsp70 in the extending region and central keloid region was quantitatively measured with MetaMorph image analysis software (Universal Image Corp.) and was statistically significant compared to the adjacent normal area. N, normal; E, extending region; K, keloid region (×200, *P < 0.001).

To evaluate the expression pattern of Hsp70 in keloid tissue, immunohistochemical staining with anti-Hsp70 monoclonal antibody was carried out. H&E staining and TGF-*β* immunohistochemical staining were also performed to see the expression pattern compare with Hsp70. Compared to normal dermis, markedly increased immunopositivity for Hsp70 was noted in keloid region. With H&E staining, we found that keloid tissue had a dense and excessive deposition of collagen in the dermis along with Hsp70 expression. Another interesting finding was extension of Hsp70 positive cells and dense collagen fiber over the clinical keloid margin (Figure 3). When obtaining keloid specimen, we excised extruding keloid mass along with 3~5 mm normal tissue. Upon histological examination even the area which looked like normal tissue on gross view showed Hsp70 positive cells along with dense collagen bundle (extended region). Among the most well-known growth factors in keloid pathogenesis, TGF-*β* expression was also increased in both keloid region and extended dermal fibrosis region (Figure 3). The increased expression of Hsp70 was quantitatively measured with MetaMorph image analysis software (Universal Image Corp.) and found to be statistically significant in both central keloid region and extending dermal fibrosis (*P* < 0.01) (Figure 4). There was no statistical significant difference between central keloid and extending region; however, it seems that Hsp70 was even more highly expressed in extending region than central keloid region. 

### 3.4. Proliferating Activity Was Significantly Increased in Keloid Fibroblasts Compared to Normal Fibroblasts

**Figure 5:**
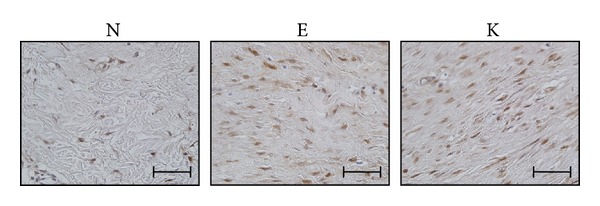
Central keloid region and histologically revealed extending area showed increased PCNA staining. N, normal; E, extending region; K, keloid region.

To evaluate proliferating activity in keloid fibroblasts, a PCNA immunohistochemical staining was performed on sections from keloid tissues. Compared to normal dermis, markedly increased immunopositivity for PCNA was noted in central keloid region and also in extended region (Figure 5). Increased PCNA expression was strongly correlated with the TGF-*β* and Hsp70 expression. 

## 4. Discussion

High-throughput screening using proteomics techniques facilitates the identification of protein interactions and allows for the analysis of differences in protein expression between keloid fibroblasts and normal fibroblasts. Using these experiments, it is possible to comprehensively understand the keloid pathogenesis [13, 14] and to find novel proteins which might have important role in abnormal fibrosis process. In this study, through proteomics technology we found 16 differentially expressed protein spots. Among these proteins, five Hsp chaperone molecules were up-regulated or down-regulated in keloid fibroblasts. Hsp70-1A, Hsp70-1L, Hsp70-5 and Hsp70-9b were up-regulated or specifically expressed in keloid fibroblasts and Hsp27 was down regulated in keloid fibroblasts. We therefore performed further evaluation for Hsp70 which is known to be highly inducible in stressful conditions and acts as a molecular chaperon which protects cells from abnormal protein aggregation [15, 16].

Our results showed consistently increased Hsp70 in keloid fibroblasts and keloid tissues. Increased Hsp70 expression was correlated with increased expression of Hsp70 and TGF-*β*. Furthermore two of four keloid tissues have collagen fibers which seem to penetrate to the adjacent normal tissue over the clinical keloid margin. 

Heat shock protein is a highly conserved protein which is expressed constantly or inducible under the various physiological and environmental stresses. Hsps have protective roles to the encountered stressors and also important regulatory roles in the control of apoptosis [17, 18]. They are classified in subgroups by their molecular weight. Recently Hsp47 has been reported to be upregulated in keloid fibroblasts and could induce excessive collagen accumulation by enhancing synthesis and secretion of collagen [19]. Hsp70 family is one of the most prominent and best characterized stress proteins [20]. Because keloid fibroblasts are under hypoxic and hypermetabolic status [7, 21–23], we hypothesized that there might be a close relationship between Hsp70 and excessive collagen accumulation in keloid formation.

Hsp70 is an important regulator in apoptosis process. It inhibits key effectors of the apoptotic cascades such as the apoptosome, the caspase activation complex, and apoptosis-inducing factor (AIF) [24, 25]. Also, it is well known that the antiapoptotic effects of Hsp70 are proposed to occur by inhibiting the release of cytochrome c from the mitochondria and preventing the activation of caspase-3 [24–26]. Recent studies have shown that keloid lesions are found to have lower rates of apoptosis (22% decrease) than normal skin [27, 28], and cultured keloid fibroblasts have decreased levels of apoptosis regulatory proteins and apoptosis itself [7, 9, 18]. Other studies have reported that Hsp70 is overexpressed in keloid tissues. Totan et al. [29] showed increased expression of Hsp27, Hsp47, and Hsp70 in keloid tissue sample, and Javad and Day [13] showed increased expression of Hsp60 and Hsp70 through a protein profiling using keloid tissue. Supporting these reports we achieved similar results from cultured fibroblasts. 

In this study, we found that Hsp70 is significantly increased in keloid and keloid tissues. Using PCNA staining, we showed increased proliferative activity of keloid fibroblasts than adjacent normal fibroblasts. Increased PCNA staining region was also highly expressed Hsp70 and TGF-*β*. Furthermore, thick collagen bundles were located along with these overexpressed staining patterns. Thus, we suggest that overexpression of Hsp70 may have an important role in keloids by protecting keloid fibroblasts from hypoxia and hypermetabolic status. 

Another interesting finding was infiltrating thick collagen bundle over the clinical keloid margin. This region also showed strong positivity with Hsp70, TGF-*β*, and PCNA staining, and it was even stronger than keloid center. Clinically, keloids tend to overgrow from original scar having a tumor-like behavior. Hyperproliferating fibroblasts with overexpressed Hsp70 and TGF-*β* might lead to this clinical characteristic of keloids. 

Hsp70 is also known to stimulate the synthesis and secretion of proinflammatory mediators such as TNF-*α*, IL-1*β*, and iNOS during wound healing process [30–32]. Furthermore, overexpression of Hsp70 in response to hypoxic injury is known to induce the expression of TGF-*β*1 isoforms [33, 34]. Cao et al. [33] reported that the overexpression of Hsp70 induces the expression of TGF-*β* isoforms in keloid fibroblasts, causing excessive synthesis and deposition of extracellular matrix. However, other investigators have reported that Hsp70 might inhibit TGF-*β* signaling, partially dependent or independent of Hsp90 activity [35]. In our study, TGF-*β* was overexpressed in keloids in the same manner as Hsp70. However, it is currently unclear whether the overexpressed Hsp70 observed in keloids is a causative factor or a consequence of overproduced TGF-*β*.

Cellular skeleton associated proteins, such as tropomyosin and vimentin, were identified from the proteome map. These proteins are involved in the stabilization of intracellular skeletal components such as actin and play roles in the maintenance of cell shape and cell migration [36, 37]. Prosomal P27K protein, which is not expressed in keloid fibroblasts, is involved in the regulation of cell differentiation and the cell cycle. Also, because of the proteolytic activity of this protein, it is involved in protein degradation [38, 39].

## 5. Conclusion

We found several Hsp70 family molecular chaperone proteins increased in keloid fibroblasts than in normal fibroblasts with proteomics analysis. Overexpression of Hsp70 in keloid fibroblasts was confirmed by western blot, RT-PCR, and immunohistochemistry. We suggested that the overexpression of Hsp70 may be involved in excessive collagen production and deposition of keloids. The possible mechanism of overexpressed Hsp70 in keloids can be categorized into three areas: (1) the apoptotic pathway, (2) proinflammatory fibroblasts cytokines and growth factors, (3) and TGF-*β*. However, in which step Hsp70 affects the keloid formation was not investigated in this study. Therefore, further investigation using neutralizing antibodies against Hsp70 or siRNA-Hsp70 will be needed and is currently being pursued.

## Figures and Tables

**Table 1:** Profiles of keloid tissues, fibroblasts, and adjacent normal fibroblasts from the same patients.

No.	Sex	Age	Origin	Others
1	M	34	Chest	Proteomics
2	F	23	Earlobe	Proteomics
3	F	22	Chest	qPCR, WB
4	F	22	Axilla	qPCR, WB
5	F	19	Shoulder	qPCR, WB
6	F	29	Earlobe	qPCR, WB
7	M	25	Neck	qPCR
8	F	12	Foot	qPCR
9	F	4	Buttock	qPCR, WB
10	F	48	Earlobe	qPCR, WB
11	F	41	Neck	qPCR, WB
12	M	10	Arm	qPCR, WB
13	F	51	Chest	IHC
14	M	71	Chest	IHC
15	M	71	Chest	IHC
16	M	71	Arm	IHC
17	F	35	Earlobe	WB

**Table 2:** Results of the qualitative and quantitative analyses of 2DE performed in keloid fibroblasts and adjacent normal fibroblasts.

Spot no.	Identified protein	Accession number	Sequence coverage (%)	Theoretical Mr (KDa)/pI	Measured Mr (KDa)/pI	Est'd Z	Characteristics
**B8**	**Heat shock 70 kDa protein 9B**	**NP-004125**	**23**	**5.7/70**	**5.9/74.02**	**1.68**	**Keloid-specific**
B12	Vimentin	A25074	20	4.9/44	5.1/53.7	1.79	Keloid-specific
**B16**	**DnaK-type molecular chaperone HSPA1L**	**A29160**	**24**	**5.7/70**	**5.4/70.21**	**2.16**	**Keloid-specific**
B19	Dihydropyrimidinase-like 2	NP-001377	20	6.5/60	6/62.84	1.67	Keloid-specific
B-IN1	Crocalbin-like protein	AAF76141	38	4.3/40	4.4/34.97	1.71	Upregulated in keloid
	Calumenin	AAK72908	36		4.4/37.26		Upregulated in keloid
B-IN2	Vimentin	NP-003371	29	4.9/45	5.1/53.74	2.3	Upregulated in keloid
B-IN3	BiP protein	AAF13605	23	5/75	5.2/71.06	2.15	Upregulated in keloid
A11	Proteasome alpha 6 subunit	NP-002782	27	6.6/28	6.3/27.95	2.37	Does not exist in keloid
A-DE1	Tropomyosin 1	A23562	28	5/40	4.6/33.04	2.34	Upregulated in keloid
**A-DE3**	**Heat shock 70 kDa protein 1A**	**NP-005336**	**27**	**5.7/70**	**5.5/70.38**	**2.32**	**Upregulated in keloid**
A-IN1	Vimentin	AAA61279	39	5/55	5/53.77	2.35	Downregulated in keloid
A-IN2	Vimentin	NP-003371	37	5/55	5.1/53.74	2.39	Downregulated in keloid
A-IN4	Vimentin	A25074	30	5.1/55	5.1/53.7	2.35	Downregulated in keloid
A-IN6	Tropomyosin 4-anaplastic lymphoma kinase fusion protein	AAK17926	12	5/40	4.9/36.78	2.06	Downregulated in keloid
A-IN11	Endoplasmic reticulum protein 29 precursor	NP-006808	28	6/29	6.8/29.05	2.09	Downregulated in keloid
**A-IN12**	**Heat shock 27 kDa protein 1**	**NP-001531**	**33**	**6.1/26**	**6/22.84**	**2.35**	**Downregulated in keloid**
